# Supplementary material for: ShinyLearner: A containerized benchmarking tool for machine-learning classification of tabular data
Source: Gigascience. 2020 Apr 6;9(4):giaa026. doi: 10.1093/gigascience/giaa026 (PMC7131989; doi:10.1093/gigascience/giaa026)
Supplement: giaa026_Response_to_Reviewer_Comments_Original_Submission [file giaa026_response_to_reviewer_comments_original_submission.pdf]

Author's Response To Reviewer Comments

Close

Reviewer #1: The manuscript by Piccolo et al presents a new tool for comparing classification algorithms in a consistent interface. A key strength of the current approach is that it uses Docker containers so that once a user has installed Docker, a wide variety of algorithms from different machine learning toolboxes can be compared. The typical user for this package would be someone new to machine learning wishing to compare a wide range of algorithms on their data.

>> Thank you for taking the time to write this review and for providing this positive feedback!

I am grateful to the authors for providing online resources; in particular the Code-Ocean capsule worked for me (after first logging in; presumably anonymous access is not possible), and all the figures from the manuscript were regenerated from the analysis results. (But if the online Rmd is to be updated, the output figures could be made taller as some of the y-axes were too squashed to see the range of data, e.g. I could not see the negative, red, differences for Figure 5 online).

>> Yes, as far as we understand it, anonymous access is not possible via Code Ocean. Thank you for the feedback about some of the figures being squashed. This is true for the HTML output file, produced from the Rmd file. However, we don't know of a way to control those dimensions. If you look at the right-hand panel of the output in Code Ocean, you should be able to see PDF files that are generated from the R code. The names of those files coincide with names specified in the Rmd file, so you should be able to match those and see what they look like in the correct dimensions.

What I couldn't do however, was to re-run the analysis.
https://github.com/srp33/ShinyLearner/blob/master/Demo/Execute_Algorithms.ipynb
lists the script, and it looks appropriate, but as I'm already late with this review, I'll have to assume it works. (How long does it take to execute?) Could it also be hosted on CODE OCEAN, or would it take too long?

>> It takes several days for this to execute. Although Code Ocean does not put hard limits on execution times, it's not really designed for such long-running executions.

The text from
https://github.com/srp33/ShinyLearner/blob/master/Word_of_Caution.md
is important and should be copied into the discussion section of the paper.

>> Thank you for this suggestion. We have added a slightly modified version of this text to the end of the Discussion section in our manuscript.

Although all the code is available on github, I think an archive of the github repo should be stored on Zenodo to give a permanent DOI of the repository when (assuming) the manuscript is published.

>> We have created Zenodo archives of the relevant GitHub repositories for this tool and have added the DOI for these archives to the manuscript under "Availability of source code and requirements".

The online tool http://bioapps.byu.edu/shinylearner/ looks great, but again due to being late with this review, I didn't get time to run it

yet for myself. It would of course help for the very first time that there is a demo where I could download some data first (e.g. the Iris dataset, or MNIST) to work through this. Am I right in assuming that the role of the GUI is to build the eventual docker command to then be run locally?

>> You are correct that the role of the GUI is to build the eventual Docker command, which would be run locally. The GUI provides a link to a page on our GitHub site (https://github.com/srp33/ShinyLearner/blob/master/InputFormats.md), which describes input formats that can be used and provides example data files.

Minor:

I found it confusing to constantly flip between the main and supplementary figures. If a figure is important, please could it be folded into the main document? The first figure reference is on line 104, and that is to S1 (showing the docker command line invocation), rather than Figure 1 of the paper.

>> In the main part of the manuscript, we included what we thought would be figures that are most interesting to readers and relegated the remaining figures to the supplementary material. It just happened that the first figure we referenced was a supplementary figure, but the order in which the figures are mentioned does not necessarily indicate the figure's importance (as with any paper). Having said that, we can see the value in including the first supplementary figure in the main body, and we have made that change. When submitting our updated manuscript, we have uploaded the figures as separate files.

line 42: SUPPORT --> REQUIRE

>> This change has been made.

Is figure 2 required? It was obvious from figure 1 (to me at least) that the HoeffdingTree and decision_tree algorithms were lagging behind the others.

>> This figure has been moved to the Supplementary Material. We feel that it will be useful to some readers, even though it overlaps somewhat with the other figure.

lines 281-284: You show here that there are a few differences between algorithms that should be working the same. Did you explore why there were small differences? Parameter settings or initialisation methods? (I'm not surprised there are small differences, but thought you could explain them.)

>> We have added the following text for clarification: "For both of these algorithms, the available hyperparameters as well as options that users can select are considerably different between the underlying machine-learning libraries."

Figure 7: what classifier was used to do this analysis?

>> We have revised the caption for this figure to indicate that all 10 classification algorithms were used and that we averaged the results across these.

In the discussion, (line 389-396), six reasons supporting use of ShinyLearner are presented. I am convinced of the first two reasons, but I think most competent programmers would feel that they could also investigate points 3--6 in their own environment. Unless of course you are arguing that only ShinyLearner provides the wide diversity of algorithms that is absent in one environment (like R or Python). However, if you are to make this case, I think you need to point out specific examples of e.g. what classes of methods (rather than implementations) are missing e.g. in R or Python. My hunch, but happy

to be proven wrong, is that R and Python each provide pretty much
close to a full toolkit of machine learning methods.

>> To address these questions, we have added the following statement to the manuscript: "Although many of these tasks could be performed by a researcher who has programming expertise, care must be taken to ensure that the steps are performed in a robust manner (e.g., not mixing training and test sets in nested validation). In addition, we hope ShinyLearner will increase the efficiency of such benchmark studies by reducing duplicate efforts."

Figure S1: it looks like you are punching holes from Docker into the
user's directory. I think you need to explain any potential security
risks here.

>> This should not be a security concern. To share data between the host operating system and the container, the user executing the container must have permission to read and write data in the host directories. Using commands such as those recommended by our GUI will ensure that the container can only read/write files for which the user has permission. There are other potential security concerns with Docker, but we feel that a discussion of those topics is outside the scope of this manuscript.

Figure S2: explain vertical dotted lines in legend.

>> We have added this to the caption.

Figure S3 (and S4): Are the Coefficients of Variation simply (s.d. / mean) or
have they been multiplied by 100 to be a percentage?

>> The Coefficient of Variation is often expressed as a percentage in academic papers. We have modified the figure captions to clarify that these are percentages.

Figure S8: what does color denote?

>> We have clarified this in the caption.

Figure S10: Took me a while to work out the three coloured curves are
for the three patients; perhaps rework last sentence of legend to make
this clearer.

>> We have clarified this in the caption.

The word "Shiny" in the title should be explained somewhere to refer
to the Shiny R package for making GUIs.

>> We have modified the text to explain this.

Stephen Eglen

Reviewer #2: The authors present a very compelling tool that allows researchers to compare and benchmark algorithms across various packages regardless of their design. Shinylearner also provides a simple web interface that allows users to easily generate Docker commands that anyone can execute (assuming they have a basic knowledge of Docker).

I found the rationale of the paper to be very clear and coherent. The authors make a clear case for the need for benchmarking, especially when comparing disparate algorithms and their various software implementations, especially from the lens of applying supervised learning to biomedical studies.

ShinyLearner is modular and extensive, and language agnostic, making it widely useful to a broad community of researchers. It also allows for diverse inputs and standardizes outputs. By running all benchmarks inside a container, it removes the challenges of dealing with complex dependencies, especially for users that may not be technically savvy, all the while not building out a black box. It supports a large number of classification algorithms, has gpu support and is already set up to be

sustainable.

>> We thank the reviewer for these positive and encouraging comments!

Close
