# Supplementary material for: ShinyLearner: A containerized benchmarking tool for machine-learning classification of tabular data
Source: Gigascience. 2020 Apr 6;9(4):giaa026. doi: 10.1093/gigascience/giaa026 (PMC7131989; doi:10.1093/gigascience/giaa026)
Supplement: giaa026_Reviewer_1_Report_Original_Submission — Stephen Eglen -- 7/15/2019 Reviewed [file giaa026_reviewer_1_report_original_submission.pdf]

Reviewer Report

Title: ShinyLearner: A containerized benchmarking tool for machine-learning classification of tabular data

Version: Original Submission **Date:** 7/15/2019

Reviewer name: Stephen Eglen

Reviewer Comments to Author:

The manuscript by Piccolo et al presents a new tool for comparing classification algorithms in a a consistent interface. A key strength of the current approach is that it uses Docker containers so that once a user has installed Docker, a wide variety of algorithms from different machine learning toolboxes can be compared. The typical user for this package would be someone new to machine learning wishing to compare a wide range of algorithms on their data.
I am grateful to the authors for providing online resources; in particular the Code-Ocean capsule worked for me (after first logging in; presumably anonymous access is not possible), and all the figures from the manuscript were regenerated from the analysis results. (But if the online Rmd is to be updated, the output figures could be made taller as some of the y-axes were too squashed to see the range of data, e.g. I could not see the negative, red, differences for Figure 5 online).
What I couldn't do however, was to re-run the analysis.
https://github.com/srp33/ShinyLearner/blob/master/Demo/Execute_Algorithms.ipynb
lists the script, and it looks appropriate, but as I'm already late with this review, I'll have to assume it works. (How long does it take to execute?) Could it also be hosted on CODE OCEAN, or would it take too long?
The text from
https://github.com/srp33/ShinyLearner/blob/master/Word_of_Caution.md
is important and should be copied into the discussion section of the paper.
Although all the code is available on github, I think an archive of the github repo should be stored on Zenodo to give a permanent DOI of the repository when (asssuming) the manuscript is published.
The online tool http://bioapps.byu.edu/shinylearner/ looks great, but again due to being late with this review, I didn't get time to run it yet for myself. It would of course help for the very first time that there is a demo where I could download some data first (e.g. the Iris

dataset, or MNIST) to work through this. Am I right in assuming that the role of the GUI is to build the eventual docker command to then be run locally?

Minor:

I found it confusing to constantly flip between the main and supplementary figures. If a figure is important, please could it be folded into the main document? The first figure reference is on line 104, and that is to S1 (showing the docker command line invocation), rather than Figure 1 of the paper.

line 42: SUPPORT --> REQUIRE

Is figure 2 required? It was obvious from figure 1 (to me at least) that the HoeffdingTree and decision_tree algorithms were lagging behind the others.

lines 281-284: You show here that there are a few differences between algorithms that should be working the same. Did you explore why there were small differences? Parameter settings or initialisation methods? (I'm not surprised there are small differences, but thought you could explain them.)

Figure 7: what classifier was used to do this analysis?

In the discussion, (line 389-396), six reasons supporting use of ShinyLearner are presented. I am convinced of the first two reasons, but I think most competent programmers would feel that they could also investigate points 3--6 in their own environment. Unless of course you are arguing that only ShinyLearner provides the wide diversity of algorithms that is absent in one environment (like R or Python). However, if you are to make this case, I think you need to point out specific examples of e.g. what classes of methods (rather than implementations) are missing e.g. in R or Python. My hunch, but happy to be proven wrong, is that R and Python each provide pretty much close to a full toolkit of machine learning methods.

Figure S1: it looks like you are punching holes from Docker into the user's directory. I think you need to explain any potential security risks here.

Figure S2: explain vertical dotted lines in legend.

Figure S3 (and S4): Are the Coefficients of Variation simply (s.d. / mean) or have they been multiplied by 100 to be a percentage?

Figure S8: what does color denote?

Figure S10: Took me a while to work out the three coloured curves are for the three patients; perhaps rework last sentence of legend to make this clearer.

The word "Shiny" in the title should be explained somewhere to refer to the Shiny R package for making GUIs.

Stephen Eglen

Level of Interest

Please indicate how interesting you found the manuscript: Choose an item.

Quality of Written English

Please indicate the quality of language in the manuscript: Choose an item.

Declaration of Competing Interests

Please complete a declaration of competing interests, considering the following questions:

- Have you in the past five years received reimbursements, fees, funding, or salary from an organisation that may in any way gain or lose financially from the publication of this manuscript, either now or in the future?
- Do you hold any stocks or shares in an organisation that may in any way gain or lose financially from the publication of this manuscript, either now or in the future?
- Do you hold or are you currently applying for any patents relating to the content of the manuscript?
- Have you received reimbursements, fees, funding, or salary from an organization that holds or has applied for patents relating to the content of the manuscript?
- Do you have any other financial competing interests?
- Do you have any non-financial competing interests in relation to this paper?

If you can answer no to all of the above, write 'I declare that I have no competing interests' below. If your reply is yes to any, please give details below.

I have received a small grant from Mozilla to develop CODECHECK system, and I have been thinking whilst reviewing that this paper could well be a template paper to work on CODECHECK.

I agree to the open peer review policy of the journal. I understand that my name will be included on my report to the authors and, if the manuscript is accepted for publication, my named report including any attachments I upload will be posted on the website along with the authors' responses. I agree for my report to be made available under an Open Access Creative Commons CC-BY license (http://creativecommons.org/licenses/by/4.0/). I understand that any comments which I do not wish to be included in my named report can be included as confidential comments to the editors, which will not be published.

Choose an item.

To further support our reviewers, we have joined with Publons, where you can gain additional credit to further highlight your hard work (see: https://publons.com/journal/530/gigascience). On publication of this paper, your review will be automatically added to Publons, you can then choose whether or not to claim your Publons credit. I understand this statement.

Yes Choose an item.
